# Transcriptomic analysis reveals that mTOR pathway can be modulated in macrophage cells by the presence of cryptococcal cells

**DOI:** 10.1590/1678-4685-GMB-2020-0390

**Published:** 2021-08-02

**Authors:** Alícia C. Piffer, Francine M. dos Santos, Marcos P. Thomé, Camila Diehl, Ane Wichine Acosta Garcia, Uriel Perin Kinskovski, Rafael de Oliveira Schneider, Alexandra Gerber, Bruno César Feltes, Augusto Schrank, Ana Tereza R. Vasconcelos, Guido Lenz, Lívia Kmetzsch, Marilene H. Vainstein, Charley C. Staats

**Affiliations:** 1 Universidade Federal do Rio Grande do Sul, Programa de Pós-Graduação em Biologia Celular e Molecular, Centro de Biotecnologia, Porto Alegre, RS, Brazil.; 2 Laboratório Nacional de Computação Científica, Petrópolis, RJ, Brazil.; 3 Universidade Federal do Rio Grande do Sul, Instituto de Informática, Porto Alegre, RS, Brazil.

**Keywords:** Macrophage, mTOR pathway, Cryptococcus, RNAseq, interatomic networks

## Abstract

*Cryptococcus neoformans* and *Cryptococcus gattii* are the etiological agents of cryptococcosis, a high mortality disease. The development of such disease depends on the interaction of fungal cells with macrophages, in which they can reside and replicate. In order to dissect the molecular mechanisms by which cryptococcal cells modulate the activity of macrophages, a genome-scale comparative analysis of transcriptional changes in macrophages exposed to *Cryptococcus* spp. was conducted. Altered expression of nearly 40 genes was detected in macrophages exposed to cryptococcal cells*.* The major processes were associated with the mTOR pathway, whose associated genes exhibited decreased expression in macrophages incubated with cryptococcal cells. Phosphorylation of p70S6K and GSK-3β was also decreased in macrophages incubated with fungal cells. In this way, *Cryptococci* presence could drive the modulation of mTOR pathway in macrophages possibly to increase the survival of the pathogen.

## Introduction

The worldwide incidence of cryptococcosis is approximately 220,000 cases per year, with mortality rates of 80% (Rajasingham *et al*., 2017). This disease is caused by the basidiomycete yeasts *Cryptococcus neoformans* and *Cryptococcus gattii*. Although *C. neoformans* and *C. gattii* show 80-90% genomic identity and share several virulence factors (D’Souza *et al*., 2011; Bielska and May, 2016), these species can affect distinct individuals with different disease manifestations. *C. neoformans* causes disease in immunocompromised patients, with a tendency to disseminate to the central nervous system. Meanwhile, *C. gattii* can affect both immunocompromised and healthy individuals, with some strains showing reduced dissemination to the central nervous system in murine models of cryptococcosis (Speed and Dunt, 1995; Ngamskulrungroj *et al*., 2012; Bielska and May, 2016; Barcellos *et al*., 2018).

The disease is initiated with the inhalation of basiodiospores or desiccated cells from environmental sources and subsequent interaction with alveolar phagocytic cells, such as macrophages, dendritic cells and neutrophils (Osterholzer *et al*., 2009; Brown, 2011). These mammalian cells engulf the pathogen and expose the yeast to damaging agents, such as low pH, reactive oxygen species, nitric oxide, as well as proteases, to kill the invading pathogen (Ghosn *et al*., 2006; Leopold Wager *et al*., 2016). However, the fungal cells are capable of reducing macrophage antifungal activity to survive and replicate inside the phagosome (Tucker and Casadevall, 2002; Alvarez and Casadevall, 2006). *C. neoformans* can prevent significant acidification of the phagosome, calcium efflux and protease activity, rendering the phagosome permissive to cryptococcal proliferation *in vitro* (Smith *et al*., 2015). In addition, cryptococcal cells drive lysosomal damage in bone marrow derived macrophages, which is correlated with increased *C. neoformans* intracellular replication (Davis *et al*., 2015). *C. gattii* fungal cells can deregulate the maturation of dendritic cells causing suboptimal T cell activation and proliferation (Huston *et al*., 2013). Moreover, such pathogenic yeasts can modulate macrophage polarization to M2 (alternatively activated), thereby evading recognition and killing by the host (Leopold Wager *et al.,* 2016). Even with the elucidation of several mechanisms showing that cryptococcal cells modulate the immune system (Leopold Wager *et al.,* 2016; Casadevall *et al.,* 2018), there are still points to be studied and clarified in this field. 

In this study, pre-activated J774.16 macrophage-like cells were co-incubated with *C. gattii* R265 and *C. neoformans* H99 fungi cells and the gene transcription profile of host cells was evaluated. Network analysis revealed that the expression of central genes associated with the mTOR signaling pathway were altered, suggesting that *Cryptococcus* spp. cells possibly modulate several bioprocesses in phagocytic cells, thereby decreasing the activation of the mTORC1 in mammalian cells. The modulation of the Akt/mTOR pathway was confirmed through the observed decrease in the phosphorylation of p70S6K and GSK-3β in macrophages incubated with fungal cells. 

## Material and Methods

### Strains and cell lines

The pathogenic yeasts, *Cryptococcus gattii* strain R265 and *Cryptococcus neoformans* strain H99, were routinely maintained in YPD agar media (2% glucose, 2% peptone, 1% yeast extract and 1.5% agar; Sigma Aldrich). The murine macrophage-like cell line, J774.16, obtained from *Banco de Células do Rio de Janeiro* (BCRJ - accession number 0273) and cultured with Dulbecco’s modified Eagle’s medium (DMEM; Gibco Life Technologies) supplemented with 10% heat-inactivated fetal bovine serum (FBS; Gibco), penicillin 20 U/mL and streptomycin 20 µg/mL (Gibco) was incubated at 37 °C with 5% CO_2_. Confluent cultures were expanded every 2 - 3 days until a maximum of 10 passages.

### Co-incubation of macrophage and fungal cells

For co-incubation assays, J774.16 macrophages were seeded in culture plates at a density of 10^6^ cells/mL and activated with 100 U/mL recombinant murine IFN-γ (Sigma Aldrich) and 500 ng/mL LPS (Sigma Aldrich) overnight. *Cryptococcus* R265 and H99 strains were grown in YPD broth (2% glucose, 2% peptone, 1% yeast extract; Sigma Aldrich) on a shaker platform at 30 °C for 18 h. After growth, cryptococcal cells were washed three times with PBS and opsonized in a density of 10^7^ cells per mL with 1 μg/mL of anti-GXM antibody 18B7 (a kind gift from Dr. Arturo Casadevall) for 1 h at 37 °C. Opsonized cryptococcal cells were incubated with previously activated J774.16 cells, with addition of 100 U/mL IFN-γ and 500 ng/mL LPS. The control samples were performed as the co-incubation assays without the addition of fungal cells. In order to achieve a maximization of macrophage-like cells containing engulfed yeast, J774.16 was activated with IFN- γ and LPS and fungal cells were opsonized (Mukherjee *et al*., 1995; Nicola and Casadevall, 2012).

### Phagocytosis assays

The determination of fungal loads in J774.16 macrophages was performed as previously described (Evans *et al*., 2017; Squizani *et al*., 2018). Briefly, cryptococcal cells previously grown in YPD for 18 h were washed with PBS, opsonized with 1 ****μg**** /mL of anti-GXM antibody 18B7, and further incubated for 30 minutes with 0.5 mg/mL of fluorescein isothiocyanate (Sigma). Fungal cells were washed with PBS and incubated with previously activated (100 U/mL IFN-γ and 500 ng/mL LPS) macrophage cells in a 12‐well culture plate and incubated for 6 h. Unattached extracellular fungal cells were washed with warm PBS and the fluorescence of labelled yeast attached to the outer membrane of macrophage was quenched by the addition of trypan blue. The phagocytosis rate was evaluated by flow cytometry (Millipore Guava‐soft) after detaching the macrophage cells with a cell scrapper. The percentage of cells with a high forward scattering signal and a high green fluorescence was considered for the determination of phagocytosis rate.

### RNA-seq assay

Activated J774.16 cells were incubated in the absence (control condition) or presence of opsonized cryptococcal cells, as described above, for further RNA extraction and transcriptome evaluation. After 6 h of co-incubation, each well was washed three times with warm PBS buffer to eliminate the macrophage-non-associated cryptococcal cells. Macrophage RNA extraction was performed with TRIzol™ (ThermoFischer Scientific) and purified with RNeasy Mini kit (Qiagen). Poly (A) RNA samples were purified using the Dynabeads® mRNA purification kit (ThermoFisher Scientific) according to the manufacturer’s instructions. The RNA quality was assessed with a Bioanalyzer 2100 system (Agilent Technologies) and sequenced in an Ion PGM System at the LNCC laboratory in Petrópolis, Rio de Janeiro in Brazil.

The data were analyzed using the FastQC software 23 and the software Fastx Toolkit (http://hannonlab.cshl.edu/fastx_toolkit/index.html) was used for sequences processing. The sequences were aligned against the *Mus musculus* genome with TMAP aligner (https://github.com/iontorrent/TMAP) and the counting was performed with HTseq program. The differentially expressed genes (DEGs) were determined using the Package TCC and EdgeR (Sun *et al*., 2013). Transcript levels were further screened by applying an unadjusted false discovery rate (FDR) of 10%. Genes with a FDR-corrected p-value < 0.05 and |log_2_ fold change| ≥ 0.58 were considered statistically significant and differentially expressed. Two major libraries were created: (i) genes that are differentially expressed in activated macrophages infected with *C. gattii* R265 compared to control (activated macrophages cultivated in the absence of fungi cells) and (ii) genes that are differentially expressed in activated macrophages infected with *C. neoformans* H99 compared to control (activated macrophages cultivated in the absence of fungi cells).

### Interatomic networks

STRING 10 was used (http://string-db.org/) (Snel *et al*., 2000; Szklarczyk *et al*., 2015) to design the interatomic networks and to elucidate the pathways involved in the macrophage response to the cryptococcal infection. DEGs found in the RNA-seq of each library was used as input data, creating two distinct networks: *C. gattii* network and *C. neoformans* network. The parameters used to prospect the networks for STRING 10 software were as follows: co-expression, experiments, databases, 700 additional nodes, no more than 20 interactions. The results generated using STRING 10 were analyzed with Cytoscape 2.8.3 (Shannon *et al*., 2003). To analyze the networks in terms of the major clusters or module composition, the Molecular Complex Detection (MCODE) program (Bader and Hogue, 2003) was used. The parameters for MCODE cluster finding were as follows: degree cutoff, 2; expansion of a cluster by one neighbor shell allowed (fluff option enabled); deletion of a single connected node from clusters (haircut option enabled); node density cutoff, 0.1; node score cutoff, 0.2; k-core, 2; and maximum network depth, 100. Centrality analysis was performed for the two main networks using CentiScaPe 1.2 (Scardoni *et al*., 2009). This analysis allowed the identification of the most topologically “central” nodes within the network using an algorithm to evaluate each node according to the node degree and betweenness. The major networks, the clusters generated by MCODE and the centrality nodes from the two major networks were further studied by focusing on major biology-associated processes using the Biological Network Gene Ontology (BiNGO) 2.44 Cytoscape 2.8.3 plugin (Maere *et al*., 2005). The degree of functional enrichment for a network was quantitatively assessed (*p*value) using hypergeometric distribution. Multiple test correction was also assessed by applying the FDR algorithm (Benjamini and Hochberg, 1995), at a significance level of *p* < 0.05.

### Quantitative real time PCR analysis

J774.16 macrophage-like cells pre-activated overnight with 100 U/mL recombinant murine IFN-γ (Sigma Aldrich) and 500 ng/mL LPS (Sigma Aldrich) were exposed to opsonized R265 and H99 cryptococcal cells for 2, 6 and 24 h and the transcription profile of some mTOR pathway genes was evaluated, *mTOR*, *Ddit4*, *Pten*, *Pdk1*, *Rictor*, *Raptor*, *Ulk1* and *TNF-α.* Control conditions were not co-incubated with yeast cells. 

After co-incubation, the wells were washed three times with warm PBS before lysis of macrophage cells with TRIzol™ reagent according to the manufacturer’s instructions. Samples were centrifuged to eliminate the non-phagocyzed cryptococcal cells and the quality of the RNA was assessed by electrophoresis on a 1% agarose gel. Quantification was performed by absorbance analysis using a NanoDrop spectrophotometer (Thermo Scientific). RNA was treated with DNase (Promega) and the cDNAs were prepared using ImProm-II™ Reverse transcriptase (Promega) using oligo-dT. RT-qPCR was performed using SYBR green (Invitrogen) on a StepOne Real-Time PCR System (Applied Biosystems), with thermal cycling conditions set with an initial step at 94 °C for 5 min, followed by 50 cycles at 94 °C for 15 s, 60 °C for 10 s, 72 °C for 15 s and 60 °C for 35 s, followed by a melting curve. All experiments were performed in biological triplicate, and each cDNA sample was also analyzed in triplicate for each primer pair. The transcript abundance was calculated using 2^-∆Ct^ (Livak and Schmittgen, 2001). The expression level of *Gapdh* gene was used as control to normalize the values across different target genes. The primers used in these analyses are listed in Table S1. Data were expressed as mean ± SEM. Statistical analyses were performed using GraphPad Prism 6 employing one-way ANOVA, followed by Tukey multi-comparison.

### Protein extraction and western blotting

J774.16 pre-activated cells were co-incubated with opsonized *C. gattii* R265 or *C. neoformans* H99 cells for 2, 6 and 24 h in 6-well culture cell plates or the pre-activated mammalian cells were incubated with 50 µg/mL of GXM polysaccharide isolated from *C. neoformans* H99 or *C. gattii* R265 for 24 h (Nimrichter *et al*., 2007). IFN-γ (100 U/mL) and LPS (500 ng/mL) was added to all samples at the same time that cryptococcal cells or GXM polysaccharide, as well as in the control, which did not contain GXM or yeast cells. After incubation, the macrophages were washed three times with warm PBS buffer, then lysed with denaturation buffer [SDS 4% (w/v), EDTA 2 mM, Tris 50 mM]. Protein extracts were incubated at 70 °C for 5 min and quantified using the Pierce™ BCA Protein Assay Kit (ThermoFisher Scientific) according to the manufacturer’s instructions. Equal amounts of protein (20 µg) were electrophoresed into SDS-polyacrylamide gels and transferred to Hybond PVDF membranes (GE Healthcare) overnight. After blocking with 10% nonfat dry milk in 1× TTBS, the membranes were incubated with the primary antibodies overnight at 4 °C and peroxidase-conjugated secondary antibodies at 4 ºC for 2 h. The signal was detected using ECL (GE Healthcare). The primary antibodies used were: phospho-p70 S6K-Thr389 (#9234; Cell signaling), phospho-GSK-3β-Ser9 (#9323; Cell signaling), total Akt (#4691; Cell signaling), and total p70S6K (sc-230; Santa Cruz Biotechnology).

## Results

### The presence of Cryptococcal cells induces changes in the gene expression profile of macrophages-like cells

To evaluate the changes that Cryptococcal cells induce in the transcriptional profile of macrophages, IFN-γ- and LPS-activated J774.16 macrophage-like cells were co-cultured with the fungal cells for 6 h before RNA-seq was performed. This period of incubation was chosen to represent the state of mammalian cells during mid-term interaction between macrophages and fungal cells, as early- and late-term responses have already been described (Coelho *et al*., 2015; Freij *et al*., 2018). In addition, the analysis of a time-point between this gap could help to elucidate the differences observed when compared the early- and late-term interaction. Counts for approximately 78% of mouse genes were detected in each condition. Using the TCC:EdgeR pipeline and considering a differentially expressed genes the ones with a FDR-corrected p-value < 0.05 and |log_2_ fold change| ≥ 0.58, 38 differentially expressed genes were detected when the mammalian cells were co-incubated with *C. gattii* R265 compared to control condition (Table S2), and 31 differentially expressed genes on co-incubation with *C. neoformans* H99 compared to control condition (Table S3). This small number of differentially expressed genes was also observed in previous studies (Coelho *et al.,* 2015; Freij *et al.,* 2018) and is possibly associated with the low fraction of macrophage cells with engulfed Cryptococci (Figure S1), as determined by flow cytometry*.* In this way, the main observed effect may be due to cryptococcal presence in the medium rather than in the intracellular compartment of macrophage cells, despite the higher phagocytosis rate of *C. neoformans* H99 compared to *C. gattii* R265 cells (Figure S1). Of these genes, three (*Ube2c*, *Kif20a*, *Iqgap3*) were upregulated in the presence of both fungi, and seven (*Ndrg1*, *Ddit4*, *Pdk1*, *Nat6*, *Pfkl*, *Hilpda*, *Bnip3*) were downregulated by co-incubation with both fungi (Tables S2 and S3). This demonstrates that even though *C. gattii* and *C. neoformans* are very similar species, they can affect the expression of distinct set of genes in this phagocytic cell line. All three genes upregulated in the presence of both fungi are related to cell cycle and proliferation. The gene *Ube2c* is necessary for degradation of mitotic cyclins (Townsley *et al*., 1997), *Kif20a* is associated with cytokinesis (Hill *et al*., 2000) and *Iqgap3* with proliferation, and capable of inducing cell-cycle re-entry when exogenously expressed in quiescent cells (Nojima *et al*., 2008). Of the genes downregulated in the presence of the yeast, three of them (*Ndrg1*, *Ddit4*, *Pdk1*) are related to Akt/mTOR pathway, a cascade involved in cell cycle progression as well in other cell functions (Finlay *et al*., 2012; Dennis *et al*., 2014; Weiler *et al*., 2014)

### The fungal cells can affect gene expression in various bioprocesses in mammalian cells

Systems biology tools were utilized to gain more information about other biological processes possibly affected in macrophages by fungal cells. First, two main networks were designed in the String 10 software using as input the differentially expressed genes identified in macrophages by the presence of *C. gattii* R265 cells against control (*C. gattii* network) and the differentially expressed genes identified in macrophages by the presence of *C. neoformans* H99 cells against control (*C. neoformans* network), followed by the addition of 700 nodes. 

These two networks were evaluated in the Cytoscape 2.8.3 software and clusters of genes in the two main networks were identified using the plugin MCODE, 8 clusters of genes in the *C. gattii* network and 12 in the *C. neoformans* network using a score cutoff of 2.0 (Table S4). For the search for genes that are more topologically central to the networks, an analysis was performed using the CentiScaPe plugin. In the network formed using data from genes identified in macrophage-like cells infected with *C. gattii*, 129 hubs bottlenecks were identified (Figure S2A), including four upregulated genes (*Bub1b*, *Ube2c*, *Tuba4a*, *Espl1*) and two downregulated genes (*Pten, Vav3*). In the network formed using data from genes identified in macrophage-like cells infected with *C. neoformans*, there were 98 hubs bottlenecks (Fig S2B) among the differentially expressed genes and just 5 upregulated genes were considered bottlenecks (*Aurka*, *Oasl1*, *Ncapd2*, *H2afx*, *Ube2c*).

**Figure 1 -:**
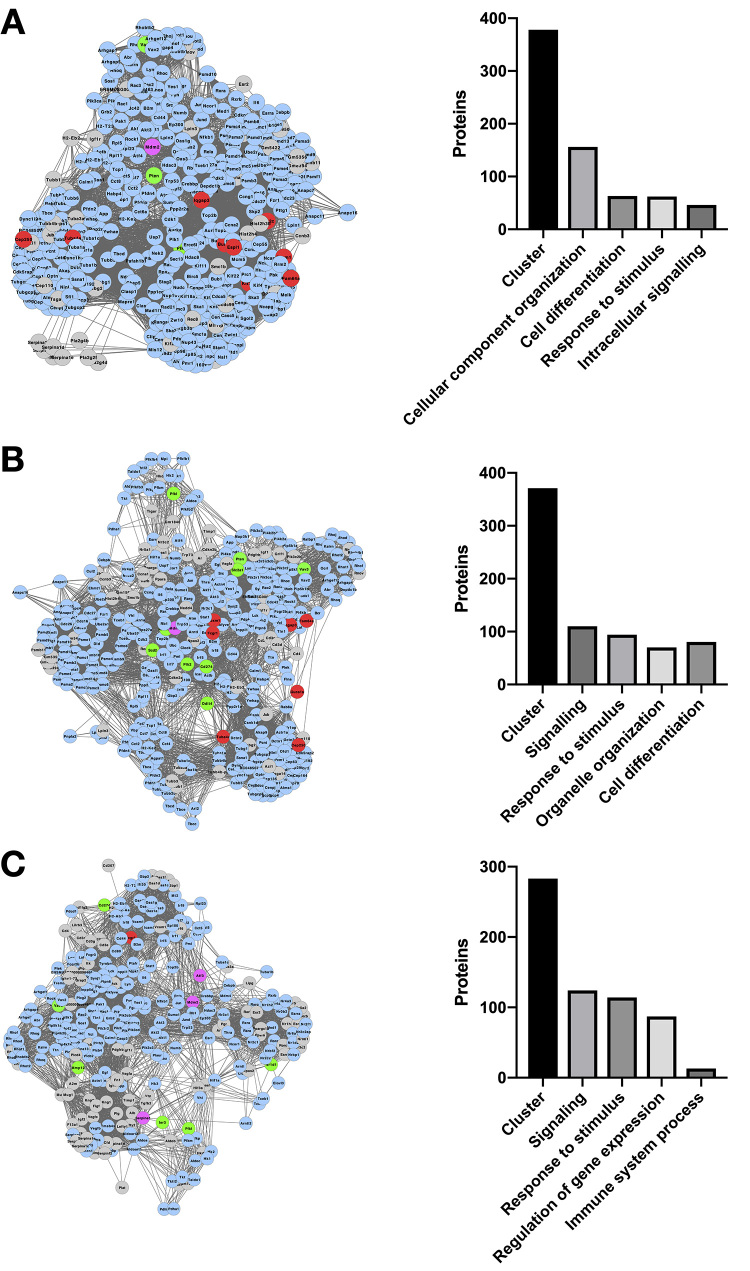
Bioprocesses potentially modulated by *C. gattii* presence in macrophage-like cells. (A-C) Enriched networks generated using the set of differentially expressed genes were clustered with MCODE. Each cluster was then analyzed for enrichment of bioprocesses using Bingo. The MCODE-generated subnetworks with the higher score are shown (left panel), as well the number of proteins present in each cluster and in the 4 bioprocesses with the highest number of associated proteins (right panel). Red: differentially expressed genes upregulated in macrophage cell line J774.16 in co-culture with fungus *C. gattii* compared to macrophage cell line without the fungi. Green: differentially expressed genes downregulated in macrophage cell line J774.16 in co-culture with *C. gattii* compared to macrophage cell line without co-incubation with yeast cells. Pink: Genes with marginal fold change (|log_2_FC|< 0.58), in macrophage cell line J774.16 in co-culture with *C. gattii* compared to macrophage cell line without co-incubation with yeast cells. Grey: genes non-detected in RNA-seq. Blue: genes were detected in the RNA-seq, but did not present differential expression in macrophage cell line J774.16 in co-culture with *C. neoformans* compared to macrophage cell line without co-incubation with yeast cells.

**Figure 2 -:**
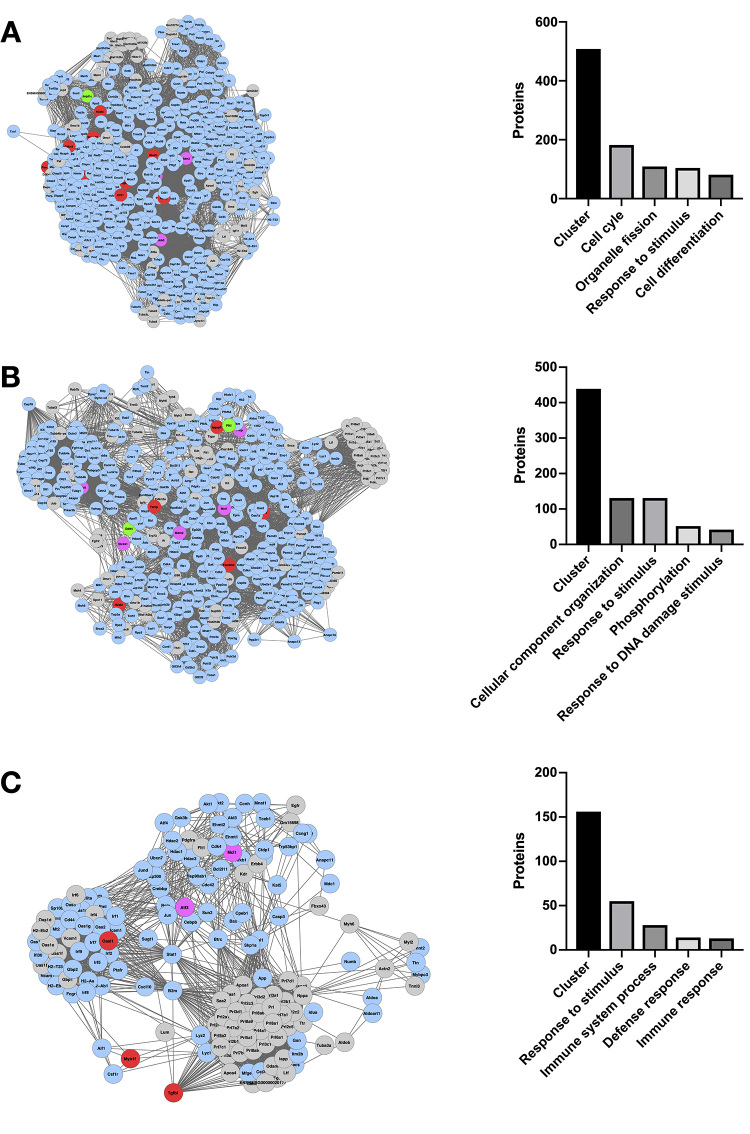
Bioprocesses potentially modulated by *C. neoformans* presence in macrophage-like cells. (A-C) Enriched networks generated using the set of differentially expressed genes were clustered with MCODE. Each cluster was then analyzed for enrichment of bioprocesses using Bingo. The MCODE-generated subnetworks with the higher score are show (left panel), as well the number of proteins present in each cluster and in the 4 bioprocesses with the highest number of associated proteins (right panel). Red: differentially expressed genes upregulated in macrophage cell line J774.16 in co-culture with fungus *C. neoformans* compared to macrophage cell line without the fungi. Green: differentially expressed genes downregulated in macrophage cell line J774.16 in co-culture with *C. neoformans* compared to macrophage cell line without co-incubation with yeast cells. Pink: Genes with marginal fold change (|log_2_FC|< 0.58), in macrophage cell line J774.16 in co-culture with *C. neoformans* compared to macrophage cell line without co-incubation with yeast cells. Grey: genes non-detected in the RNA-seq. Blue: genes were detected in RNA-seq, but did not present differential expression in macrophage cell line J774.16 in co-culture with *C. neoformans* compared to macrophage cell line without co-incubation with yeast cells.

Finally, cellular processes related to the genes present in the two main networks and clusters were analyzed using the BINGO plugin, choosing the processes who present, at least, one DEG. Besides the low similarities in genes identified in the RNA-seq data, the bioprocesses probably modulated by the presence of fungal cells are very similar. In particular, mammalian cells processes observed in the presence of *C. gattii* include signaling, cell differentiation and regulation of immune system processes (Figure 1, Table S5), while mammalian cells processes observed in the presence of *C. neoformans* condition include response to stimulus, response to DNA damage stimulus, cell differentiation and cell death (Figure 2, Table S6). Processes involved in DNA damage and response to DNA damage were uniquely found in the network formed using data from genes identified in macrophage-like cells infected with *C. neoformans*.

### Fungal cells modulate the expression level of genes involved in the mTOR pathway

**Figure 3 -:**
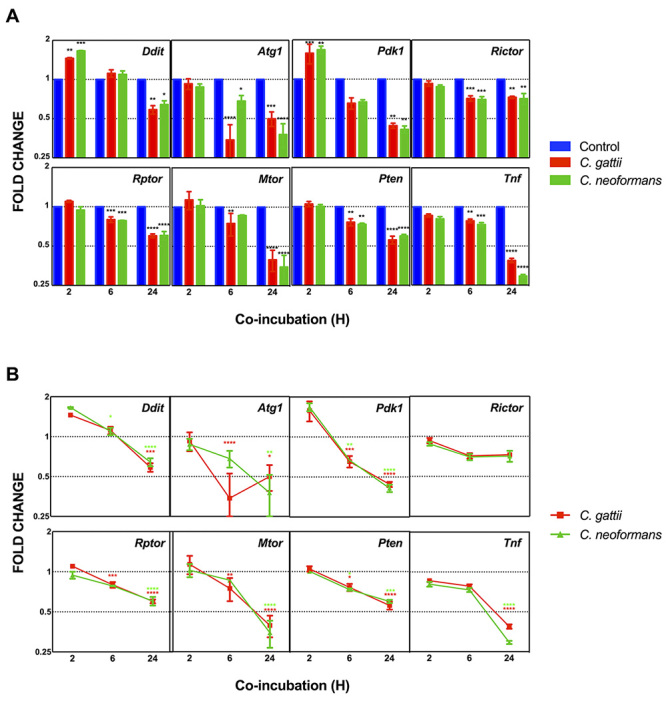
Fungal cells modulate the transcript levels of genes involved in the mTOR pathway. IFN-γ and LPS activated J774.16 cells were incubated for 2 h, 6 h and 24 h with *C. neoformans* or *C. gattii*. The measured quantity of the genes tested in each sample was normalized using the Ct values obtained for the *Gapdh* gene. Data are shown as the mean ± SEM from three experimental replicates of three biological replicates each. Columns denoted by asterisks display statistically significant differences when compared to control in each time point (A), or to the period of 2 h of co-incubation (B), as evaluated using ANOVA. *, P < 0.05; **, P < 0.01; ***, P < 0.001; ****, P < 0.0001. Red asterisks refer to the comparison between means of *C. gattii* conditions, while green asterisks refer to the comparison between means of *C. neoformans* conditions.

The comparison of the potentially modulated pathways by cryptococcal cells presence using the KEGG pathways (Kanehisa *et al*., 2017) allowed us to pinpoint a possible conserved cryptococcal influence on the macrophage Akt/mTOR pathway. This hypothesis was also supported by the detection of differentially expressed genes in the RNA-seq (*Ddit 4*, *Pdk1* and *Pten*) known to be associated with Akt/mTOR pathway (Qi *et al*., 2015; Zhang *et al*., 2018; Jhanwar-Uniyal *et al*., 2019). So, to confirm the data found in the RNA-seq and to gain information on the possible modulation in the mTOR pathway induced by fungal presence, total RNA was extracted from IFNγ- and LPS-primed macrophage-like cells after different periods in the absence (control condition) or in the presence of *C. gattii* R265 or *C. neoformans* H99 for transcriptional profiling evaluation of genes associated with the Akt/mTOR cascade (*Ddit 4*, *mTOR*, *Pdk1*, *Pten*, *Raptor*, *Rictor*, *Ulk1*, *TNF-α*), among these three were found differentially expressed in the RNA-seq (*Ddit 4, Pdk1 and Pten*). Comparison of the three different conditions in the same period of co-incubation, revealed an agreement between expression levels for the data obtained with RT-qPCR and RNA-Seq for most of the genes analyzed. Transcript levels analysis of the gene *Pdk1* and *Pten* confirmed the previously observed reduced expression in the presence of both fungi after 6 h of incubation compared with the control condition; a similar pattern was also observed after 24 h (Figure 3A). However, expression analysis of *Ddit 4* did not confirm the RNA-seq results, showing lower levels in the presence of the fungi cells after 24 h of analysis (Figure 3A), possibly as a consequence of the FDR value set at 0.1. All other genes assayed showed reduced transcript levels after 24 h incubation with both cryptococcal cells. The genes, *Rictor* and *Raptor,* showed decreased transcript levels after 6 h incubation with both fungal species, with *TNF-α* being the only gene that presented reduced transcript levels in macrophage-like cells after 2 h incubation. The gene *mTOR* was also downregulated after 6 h of incubation, but only with *C. gattii* cells (Figure 3A). In addition, the analysis of the same data in a time-course fashion showed that the presence of both fungi led to a decreased transcription level of all genes, at least after 24 h of co-incubation, except *Rictor* (Figure 3B). It is noteworthy that *C. gattii* cells caused a statistically significant decrease in the steady-state transcript levels of the evaluated genes in the macrophages, observed at 6 h of co-incubation and maintained or even decreased after 24 h of co-incubation compared to 2 h. The same pattern was observed for *Pten, Pdk1* and *Ddit* in macrophages exposed to *C. neoformans.* A statistically significant decrease in the other host genes could be only observed after 24 h of co-incubation of macrophage with *C. neoformans* cells. These data suggest that cryptococcal cells presence can modulate the Akt/mTOR pathway in IFNγ- and LPS-primed J774.16 cells.

### *C. gattii* R265 and *C. neoformans* H99 presence alters the phosphorylation levels of proteins involved in the Akt/mTOR pathway

**Figure 4 -:**
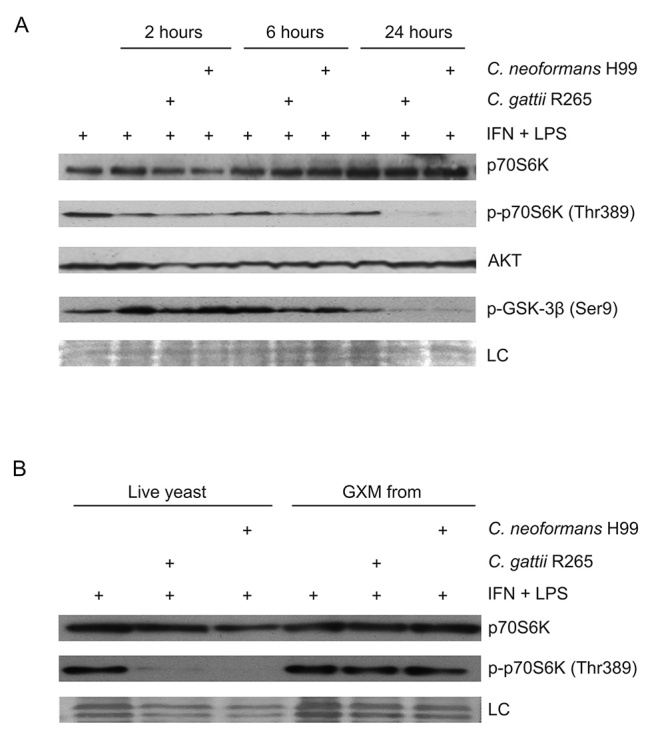
Fungal cells decreased the levels of the phosphorylated proteins p70S6K and GSK-3β. **(**A) Proteins were extracted from pre-activated J774.16 macrophage-like cells and pre-activated J774.16 macrophage-like cells after co-incubation for different times with *C. gattii* R265 or *C. neoformans* H99 and analyzed by western blotting. (B) Proteins were extracted from pre-activated J774.16 macrophage-like cells and pre-activated J774.16 macrophage-like cells after co-incubation for 24 hours with *C. gattii* R265, *C. neoformans* H99, purified R265 GXM and purified H99 GXM and subjected to western blotting. LC: loading charge.

Next, we evaluated if the modulation of mTOR pathway genes in macrophages by the presence of cryptococcal cells could also be detected at protein level or phosphorylation state. For that, proteins of J774.16 cells after 2, 6 and 24 h co-incubation with *C. gattii* R265 or *C. neoformans* H99 cells were isolated and the total and phosphorylation levels of pivotal proteins of the Akt/mTOR pathway, p70S6K, GSK-3β and Akt, were analyzed by western blotting. After 24 h co-incubation, the phosphorylation levels of p70S6K (a target of mTORC1, also known as S6K) and GSK-3β (a target of Akt) were reduced in comparison to the control (Figure 4A), with no difference in the total amount of p70S6K and Akt protein detected in extracts of macrophages incubated or not with the yeast cells.

As this modulation was observed in the co-incubation with cryptococcal cells, we asked if this regulation in the phosphorylation of p70S6K could also be observed when J774.16 cells where incubated with GXM, the most abundant capsule polysaccharide of the yeast (Zaragoza *et al*., 2009). No difference in the phosphorylation of the p70S6K in macrophages treated with *C. gattii* R265 nor *C. neoformans* H99 GXM in comparison to the control sample was observed (Figure 4B).

These data suggest that the presence of live cryptococcal cells, but not fractions of the polysaccharide capsule, cause modulation of post-translational levels in the macrophage-like cells, thereby influencing the signaling of downstream proteins. It is possible that the modulation is only caused in the presence of fungal cells, once the major capsule polysaccharide is incapable of impairing the p70S6K phosphorylation.

## Discussion

During cryptococcal infection, yeast cells develop strategies to overcome macrophage antifungal activity in order to reside and proliferate inside mammalian cells (Levitz *et al*., 1999). Considerable evidence suggests that these phagocytic cells can be strongly affected by the presence of the fungus, ultimately undergoing apoptosis (Alvarez and Casadevall, 2007; Ben-Abdallah *et al*., 2012; Coelho *et al*., 2012). In this study, a genome-scale comparative analysis of transcriptional changes in IFNγ- and LPS-primed J774.16 cells exposed to *C. neoformans* and *C. gattii* was performed to understand the processes and pathways that fungi cells can modulate in the phagocytic cells, as well as the differences that distinct fungal species can cause in this model. We used a condition in which macrophage cell were activated with IFNγ- and LPS in order to increase phagocytosis (Mukherjee *et al*., 1995; Nicola and Casadevall, 2012).

In total, 38 and 31 genes were found to be modulated by the presence of *C. gattii* and *C. neoformans*, respectively, in IFNγ- and LPS-primed J774.16 cells, which is in agreement with previous studies. Some differences were found in gene number and identity when comparing the set of genes differentially expressed in macrophages due the presence of each cryptococcal species. Despite the lower uptake of *C. gattii* R265 strain compared to *C. neoformans* H99, the majority of phagocytes did not harbor yeast cells. Therefore, we hypothesize that such effects in macrophages are caused by the presence of yeast cells in the culture medium. Coelho and coworkers used hybridization to cDNA microarrays to show that the number of differentially expressed genes in J774.16 was 35 after 2 h of incubation with cryptococcal cells, which increased to 97 after 24 h of incubation (Coelho *et al.,* 2015). Studies using human monocytes and BMDM cells were able to find much more DEGs in response to cryptococcal infection (Chen *et al*., 2015; Freij *et al*., 2018) Probably, three major differences account for these differences: (i) the use of a cell line instead of primary cells, (ii) the time of co-incubation between mammalian and fungi cells and (iii) the macrophage pre-activation. The macrophage-like cells in the present study were previously incubated with IFN-γ and LPS, a condition not used in previous works. Activation with IFN-γ and LPS polarizes the cells to a M1 phenotype (Beutler and Rietschel, 2003; Mantovani *et al*., 2004; Bryant *et al*., 2010), which could account for the differences in the differentially expressed genes identified in these studies. However, it should be noted that during mice lung infection by cryptococcal cells, cytokine expression changes dynamically over time. Therefore, both M1 and M2 phenotypes could be present concurrently during fungal infection (Arora *et al*., 2011), and the results presented here possibly constitute a fraction of the differentially expressed genes that would be observed in mice lungs infected with cryptococcal cells.

To understand the processes that could be modulated in the M1 macrophages-like cells by the fungal cells, a Gene Ontology analysis was performed on the networks with at least one DEG. Comparing the results obtained from the two networks, the biological processes modulated were similar, even with few shared differentially expressed genes. This lack of difference observed between the two species analyzed was also found before (Freij *et al*., 2018)*.* In this study, conducted in BMDM, they compared macrophage incubation with different strains of *C. gattii* and *C. neoformans,* with no strikingly difference observed. The transcriptional data provided showed a similarity of 30-50% of macrophage genes modulated by *C. neoformans* and *C. gattii* and the analyses of clustering maps revealed a stronger clustering by time of infection than by strain, suggesting that the genetic modulation induced in macrophages infected by *C. neoformans* and *C. gattii* is similar. Also, there was similarity between the modulated processes with those identified by Coelho *et al*. (2015). The bioprocesses modulated were mainly associated with the cell cycle, cell differentiation, cell death, signaling and cell communication, response to stimulus, protein modification, immune process, and glucose metabolism, of which most have been previously reported in the literature as being modulated by *C. neoformans* (Wright *et al*., 2002; Monari *et al*., 2008; Ben-Abdallah *et al*., 2012; Coelho *et al.,* 2012). However, when comparing the bioprocesses described here with those observed in human monocytes, a greater difference is observed. The study conducted by Chen *et al*. (2015) pointed to a greater modulation of genes and processes related to the immune response. This could be explained by two facts already mentioned above: the use of primary cells and non-polarization of macrophages.

It was observed that some of the modulated bioprocesses, as well as some important differentially expressed genes, are related to the Akt/mTOR pathway. The mammalian target of rapamycin (mTOR) is a conserved serine/threonine kinase that senses the external and internal signals to control numerous processes, including those related to the immune response, cell cycle and cellular death (Cornu *et al*., 2013; Katholnig *et al*., 2013; Shimobayashi and Hall, 2014; Weichhart *et al*., 2015). This protein is found in two multiprotein complexes designated mTORC (mTOR complex) 1 and mTORC2. Raptor (regulatory associated protein of mTOR) is the main regulatory protein for mTORC1, with Rictor (rapamycin-insensitive companion of mTOR) and Sin1 being specific regulators of mTORC2 (Laplante and Sabatini, 2012; Cornu *et al.,* 2013). The results presented here confirmed that this key pathway is modulated in macrophages by the presence of cryptococcal cells as (i) the expression of selected genes related to the Akt/mTOR pathway and (ii) the phosphorylation of Akt/mTOR target proteins were both altered by the presence of cryptococcal cells. These findings suggest that the mTOR pathway activity is reduced in macrophages-like cells by the presence of the yeast cells.

The Akt/mTOR pathway plays a central role in cell homeostasis and is also involved in the defense response. The mTOR complexes, especially mTORC1, are involved in the regulation of dendritic cell development, NK cell activation and proliferation, pro- and anti-inflammatory cytokine production in various cell types, macrophage polarization and nitric oxide production (Cheekatla *et al*., 2012; Katholnig *et al*., 2013; Amiel *et al*., 2014; Sukhbaatar *et al*., 2016; Vangan *et al*., 2016; Viel *et al*., 2016). In this way, it is feasible to hypothesize that cryptococcal cells presence can inhibit this pathway in order to decrease the M1 macrophage fungicidal activity. The present study provided evidence that the presence of *C. neoformans* and *C. gattii* cells drives the inhibition of mTORC1 and Akt targets phosphorylation. Activation of the Akt/mTOR pathway mediates signals from receptors such as pathogen-associated molecular pattern receptors and cytokines receptors, in a pathway involved in macrophage polarization (Katholnig *et al.,* 2013; Weichhart *et al*., 2015; Vergadi *et al*., 2017). In this work, the yeast cells inhibit Akt and mTORC1 activation in IFN-γ- and LPS-primed macrophage-like cells, suggesting that the macrophages incubated with cryptococci display M2 polarized phenotypes. This assumption corroborates previous data, which demonstrated that *C. neoformans* polarized macrophages for alternative (M2) differentiation (Müller *et al*., 2007; Arora *et al*., 2011; Eastman *et al*., 2015), and cells lacking the mTOR inhibitor, TSC1, are characterized by the constitutive mTORC1 activation and reduced M2 polarization induced by IL4 (Byles *et al*., 2013).

Macrophages polarized to M1 secrete pro-inflammatory molecules such as IFN-β, IL-12, TNF-α, IL-6, IL-1β and NO (Martinez and Gordon, 2014). The presence of the fungal cells led to reduced expression of *TNF-α* gene in IFNγ- and LPS-primed J774.16 cells. TNF-α is a pivotal pro-inflammatory cytokine involved in host defense against cryptococcal species and expression of *TNF-α* is increased in *TSC1* knockout cells (Kawakami *et al*., 1996; Shoham *et al*., 2001). As this gene product is an inhibitor of the mTOR pathway (Pan *et al*., 2012), we hypothesize that the decreased expression of the *TNF-α* gene observed in IFNγ- and LPS-primed macrophage-like cells exposed to *Cryptococcus* spp. is associated with reduced mTORC1 complex activity.

Reduced levels of activated p70S6K (p-p70S6K) were detected in this study, corroborating the downregulation in the mTORC1 pathway due to the presence of cryptococcal cells. The parasite *Leishmania major* produces GP63, a protein that is capable of proteolytically degrading mTORC1. As a consequence of reduced mTORC1 levels, macrophages display decreased global cellular translation and mRNA levels of *IFNG* and *iNOS,* that in turn, allow *L. major* to survive inside immune cells (Jaramillo *et al*., 2011). In addition, the p70S6K protein negatively regulates the expression of some M2 genes (Warren *et al*., 2016) and TIPE2 precludes M1 polarization by impeding the mTORC1 response (Jiang *et al*., 2018). Hence, it is feasible to assume that cryptococcal cells employ the inhibition of the mTORC1 complex to survive inside macrophages.

**Figure 5 -:**
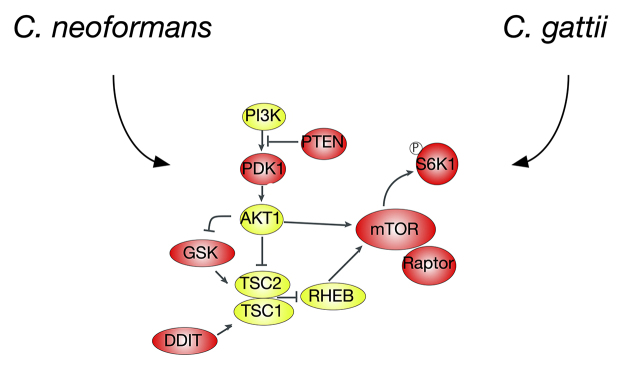
mTOR related targets of cryptococcal presence on macrophage cells. A partial mTOR pathway encompassing the analyzed genes and proteins is shown. Green labels represent proteins not evaluated. Red labels represent genes or proteins in which expression or activation are reduced by the presence of cryptococcal cells.

Intriguingly, the downregulation of two genes related to the Akt/mTOR pathway (*Pten* and *Pdk1*) was detected in this study, whose products act in an opposing way in the same substrate. PDK1 and Akt interacts with phosphatidylinositol (3,4,5)-trisphosphate (PIP3) in the plasma membrane, enabling PDK1 to phosphorylate and activate Akt. However, PTEN converts PIP3 to PIP2, inhibiting the activation of Akt by PDK1. In this way, PTEN is considered an inhibitor of Akt activation (Katholnig *et al*., 2013). As the mTOR pathway is a general regulator of several processes, it is assumed that it can also regulate the expression of proteins with antagonistic functions, as PTEN and PDK1. When considering the results here shown, globally, the downregulation of activators coding genes was more pronounced than the downregulation of inhibitors coding genes. In this way, it is feasible to assume that the final outcome could be provided by the sum of individual activities of each activator and inhibitor. Thus, the differential expression induced by the fungi could indicate a modulation of mTOR pathway (Figure 5).

In summary, the results confirm that the presence of *C. neoformans* and *C. gattii* can modulate some processes in macrophages and that despite the differences between these two species, the processes that they modulate in phagocytic cells are very similar. In this context, the inhibition of the Akt/mTOR pathway emerged as a probable key modulator of the outcomes observed after the co-incubation of macrophage cells and cryptococcal cells. Hence, direct or indirect inhibition of Akt/mTOR pathway could be used by cryptococcal cells to reduce macrophage antifungal activities.

## Supplementary Material

The following online material is available for this article:

Figure S1 - Analysis of cryptococcal cells uptake by J774.16 macrophages.

Figure S2 - Bottleneck hubs found using the set of differentially expressed genes in macrophages exposed to *C. gattii* (A) or *C. neoformans* (B) compared to control condition.

Table S1 - Primers used in this study.

Table S2 - Differentially expressed genes in J774.16 macrophage-like cells after exposure to *C. gattii*.

Table S3 - Differentially expressed genes in macrophages cells after exposure to *C. neoformans*.

Table S4 - Score and number of nodes for the two main networks.

Table S5 - Bioprocess *C. gattii* network.

Table S6 - Bioprocess *C. neoformans* network.
