# Supplementary material for: Transcriptomic analysis reveals that mTOR pathway can be modulated in macrophage cells by the presence of cryptococcal cells
Source: Genet Mol Biol. 2021 Aug 2;44(3):e20200390. doi: 10.1590/1678-4685-GMB-2020-0390 (PMC8341293; doi:10.1590/1678-4685-GMB-2020-0390)
Supplement: Figure S1 - [file 1415-4757-GMB-44-3-e20200390-s1.pdf]

Genetics and Molecular Biology
https://doi.org/10.1590/1678-4685-GMB-2020-0390

# Supplementary Material to "Transcriptomic analysis reveals that mTOR pathway can be modulated in macrophage cells by the presence of cryptococcal cells"

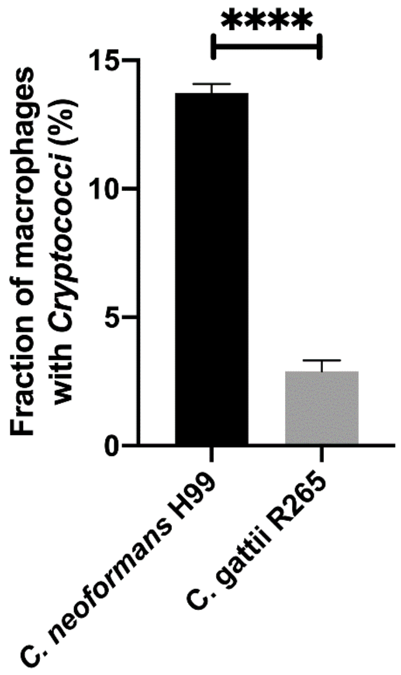


**Figure S1** - Analysis of cryptococcal cells uptake by J774.16 macrophages. The bars represents the percentage of cells with high FITC fluorescence and high forward scattering signals, representing the fraction of macrophages that harbors cryptococcal cells. Data is presentes as means of three biological replicates and standard deviation. ****, $P < 0.0001$ according to t-student test.
