# Supplementary material for: Transcriptomic analysis reveals that mTOR pathway can be modulated in macrophage cells by the presence of cryptococcal cells
Source: Genet Mol Biol. 2021 Aug 2;44(3):e20200390. doi: 10.1590/1678-4685-GMB-2020-0390 (PMC8341293; doi:10.1590/1678-4685-GMB-2020-0390)
Supplement: Figure S2 - [file 1415-4757-GMB-44-3-e20200390-s2.pdf]

Genetics and Molecular Biology
https://doi.org/10.1590/1678-4685-GMB-2020-0390

# Supplementary Material to “Transcriptomic analysis reveals that mTOR pathway can be modulated in macrophage cells by the presence of cryptococcal cells”

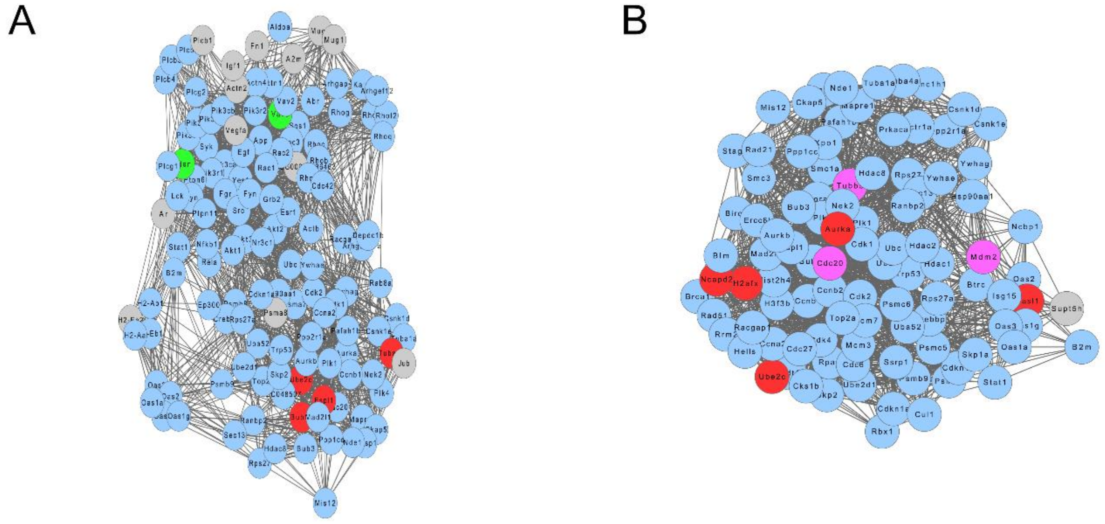


**Figure S2 -** Bottleneck hubs found using the set of differentially expressed genes in macrophages exposed to *C. gattii* (A) or *C. neoformans* (B) compared to control condition.
