# Supplementary material for: Transcriptomic analysis reveals that mTOR pathway can be modulated in macrophage cells by the presence of cryptococcal cells
Source: Genet Mol Biol. 2021 Aug 2;44(3):e20200390. doi: 10.1590/1678-4685-GMB-2020-0390 (PMC8341293; doi:10.1590/1678-4685-GMB-2020-0390)
Supplement: Table S1 - [file 1415-4757-GMB-44-3-e20200390-s3.pdf]

Genetics and Molecular Biology
https://doi.org/10.1590/1678-4685-GMB-2020-0390

Supplementary Material to "Transcriptomic analysis reveals that mTOR pathway can be modulated in macrophage cells by the presence of cryptococcal cells"

Table S1 - Primers used in this study.

Gene	Forward (5' → 3')	Reverse (5' → 3')
Ddit 4	CAAGGCAAGAGCTGCCATAG	CCGGTACTTAGCGTCAGGG
mTOR	ACCGGCACACATTTGAAGAAG	CTCGTTGAGGATCAGCAAGG
Pdk1	GTGCCCATTCAGTCCAGTGT	AAGGGGTTGGTGCTTGGTC
Pten	TGGATTCGACTTAGACTTGACCT	GCGGTTTCATAATGTCTCTCAG
Raptor	TTTGTCTACGACTGTTCCAATGC	GCTACCTCTAGTTCCTGCTCC
Rictor	GCTGCGCTATCTCATCCAAGA	GGGTTCTGAAGTGCTAGTTCAC
Ulk1	AAGTTCGAGTTCTCTCGCAAG	CGATGTTTTCGTGCTTTAGTTCC
TNF-α	AGCCTCTTCTCATTCCTGC	GGAGGCCATTTGGGAACT
Gapdh	AGGTCGGTGTGAACGGATTTG	TGTAGACCATGTAGTTGAGGTCA
