# Supplementary material for: Transcriptomic analysis reveals that mTOR pathway can be modulated in macrophage cells by the presence of cryptococcal cells
Source: Genet Mol Biol. 2021 Aug 2;44(3):e20200390. doi: 10.1590/1678-4685-GMB-2020-0390 (PMC8341293; doi:10.1590/1678-4685-GMB-2020-0390)
Supplement: Table S2 - [file 1415-4757-GMB-44-3-e20200390-s4.pdf]

Genetics and Molecular Biology
https://doi.org/10.1590/1678-4685-GMB-2020-0390

Supplementary Material to “Transcriptomic analysis reveals that mTOR pathway can be modulated in macrophage cells by the presence of cryptococcal cells”

Table S2 - Differentially expressed genes in J774.16 macrophage-like cells after exposure to *C. gattii*.

Gene	Gene_ID	Log2FC	P value	FDR-corrected P value
Diacylglycerol O-acyltransferase 2	Dgat2	1.6568	6.42E-05	0.0321
Cell division cycle associated 2	Cdca2	1.6185	3.69E-05	0.0239
Transmembrane protein 229B	Tmem229b	1.2908	1.23E-04	0.0465
IQ motif containing GTPase activating protein 3	Iqgap3	1.0776	8.04E-05	0.0357
Family with sequence similarity 64, member A	Fam64a	1.0432	5.03E-05	0.0286
Forkhead box M1	Foxm1	1.0297	2.15E-05	0.0145
Fc receptor, IgG, high affinity I	Fcgr1	1.0210	1.91E-05	0.0135
Kinesin family member 20[a]	Kif20a	0.9988	1.24E-05	0.0107
Nfat activating molecule with ITAM motif 1	Nfam1	0.9539	7.61E-05	0.0348
Extra spindle pole bodies 1, separase	Espl1	0.9133	3.79E-06	0.0042
G protein-coupled receptor 84	Gpr84	0.9053	5.64E-05	0.0302
Ubiquitin-conjugating enzyme E2C	Ube2c	0.7916	4.62E-05	0.0276
Centrosomal protein 250	Cep250	0.7896	1.43E-05	0.0117
Tubulin, alpha 4A	Tuba4a	0.7817	1.25E-08	3.25E-05

Gene	Gene_ID	Log2FC	P value	FDR-corrected P value
Guanylate cyclase activator 1a	Guca1a	0.7142	1.03E-04	0.0407
BUB1 mitotic checkpoint serine/threonine kinase B	Bub1b	0.6168	1.04E-04	0.0407
Brain abundant, membrane attached signal protein 1	Basp1	-0.5886	2.48E-06	0.0030
Stearoyl-Coenzyme A desaturase 2	Scd2	-0.6021	1.05E-04	0.0407
N-myc downstream regulated gene 1	Ndrg1	-0.7039	1.72E-05	0.0127
Proline-rich nuclear receptor coactivator 1	Pnrc1	-0.7103	3.96E-05	0.0246
CD274 antigen	Cd274	-0.7225	9.46E-05	0.0397
Polo-like kinase 2	Plk2	-0.7751	4.51E-06	0.0048
Family with sequence similarity 162, member A	Fam162a	-0.7803	5.08E-07	8.77E-04
Egl-9 family hypoxia-inducible factor 1	Egln1	-0.7889	6.87E-05	0.0333
solute carrier family 2 (facilitated glucose transporter), member 1	Slc2a1	-0.8117	4.87E-09	1.51E-05
DNA-damage-inducible transcript 4	Ddit4	-0.8485	1.69E-09	6.55E-06
Immediate early response 3	Ier3	-0.8994	1.33E-09	6.55E-06
Phosphatase and tensin homolog	Pten	-0.9014	1.68E-05	0.0127
Pyruvate dehydrogenase kinase, isoenzyme 1	Pdk1	-0.9299	6.41E-06	0.0058
Phosphofructokinase	Pfkl	-0.9369	5.61E-10	4.35E-06

Gene	Gene_ID	Log2FC	P value	FDR-corrected P value
Hypoxia inducible lipid droplet associated	Hilpda	-1.0917	7.79E-11	1.21E-06
Matrix metallopeptidase 12	Mmp12	-1.3546	5.69E-08	1.26E-04
Vav 3 oncogene	Vav3	-1.5756	7.36E-05	0.0346
N-acetyltransferase 6	Nat6	-1.6466	8.64E-08	1.68E-04
Nuclear receptor subfamily 1, group D, member 1	Nr1d1	-1.6601	9.27E-05	0.0397
1-acylglycerol-3-phosphate O-acyltransferase 9	Agpat9	-1.6832	6.01E-05	0.0311
BCL2/adenovirus E1B interacting protein 3	Bnip3	-3.1972	1.53E-06	0,0022
Ankyrin repeat domain 37	Ankrd37	-3.9218	1.22E-06	0.0019

List of the DEGs in macrophage cells after exposure to *C. gattii*. m.value: numeric vector of fold-change on log_2 scale for each gene between the two groups compared (*C. gattii* x control). p.value: numeric vector of p-value. q.value: numeric vector of q-value calculated based on the p-value using FDR of 0.1.
