# Supplementary material for: Transcriptomic analysis reveals that mTOR pathway can be modulated in macrophage cells by the presence of cryptococcal cells
Source: Genet Mol Biol. 2021 Aug 2;44(3):e20200390. doi: 10.1590/1678-4685-GMB-2020-0390 (PMC8341293; doi:10.1590/1678-4685-GMB-2020-0390)
Supplement: Table S3 - [file 1415-4757-GMB-44-3-e20200390-s5.pdf]

Genetics and Molecular Biology
https://doi.org/10.1590/1678-4685-GMB-2020-0390

Supplementary Material to "Transcriptomic analysis reveals that mTOR pathway can be modulated in macrophage cells by the presence of cryptococcal cells"

Table S3 - Differentially expressed genes in macrophages cells after exposure to *C. neoformans*.

Gene	Gene_ID	Log2FC	P value	FDR-corrected P value
Cyclin-dependent kinase inhibitor 3	Cdkn3	2.9350	8.74E-05	0.0397
Family with sequence similarity 83, member D	Fam83d	1.1525	2.55E-05	0.0181
Myosin IF	Myo1f	1.0411	8.78E-05	0.0397
Centromere protein F	Cenpf	0.9470	9.83E-05	0.0411
Kinesin family member 11	Kif11	0.8688	6.52E-05	0.0379
Ubiquitin-conjugating enzyme E2C	Ube2c	0.8326	5.02E-08	1.02E-04
Kinesin family member 20[a]	Kif20a	0.8322	9.73E-05	0.0411
IQ motif containing GTPase activating protein 3	Iqgap3	0.8090	9.84E-05	0.0411
Aurora kinase A	Aurka	0.7523	7.58E-05	0.0394
Thioredoxin interacting protein	Txnip	0.7328	9.93E-09	5.39E-05
H2A histone family, member X	H2afx	0.7296	4.37E-06	0.0059
Non-SMC condensin I complex, subunit D2	Ncapd2	0.6435	1.42E-05	0.0115
2'-5' oligoadenylate synthetase-like 1	Oasl1	0.5970	2.78E-05	0.0189
Transforming growth factor, beta induced	Tgfbi	0.5888	4.90E-06	0.0061
N-myc downstream regulated gene 1	Ndrg1	-0.5982	2.02E-05	0.0150
DNA-damage-inducible transcript 4	Ddit4	-0.5995	7.75E-05	0.0395

Gene	Gene_ID	Log2FC	P value	FDR-corrected P value
Jumonji domain containing 1C	Jmjd1c	-0.6900	7.57E-05	0.0395
Prolyl 4-hydroxylase, alpha polypeptide I	P4ha1	-0.7160	3.22E-08	8.75E-05
Elongation factor RNA polymerase II	Ell	-0.7476	1.37E-05	0.0115
A kinase (PRKA) anchor protein 2	Akap2	-0.7586	8.18E-06	0.0095
Lysine (K)-specific demethylase 3A	Kdm3a	-0.8236	4.81E-07	7.84E-04
Phosphoinositide-dependent kinase 1	Pdk1	-0.8477	2.72E-08	8.75E-05
N-acetyltransferase 6	Nat6	-0.8567	1.33E-05	0.0115
Phosphofructokinase, liver, B-type	Pfkl	-0.9725	2.03E-14	1.78E-10
Suppression of tumorigenicity 7-like	St7l	-0.9860	1.08E-05	0.0112
Mir6236 microRNA 6236	Mir6236	-1.0482	3.11E-08	8.75E-05
Hypoxia inducible lipid droplet associated	Hilpda	-1.0911	2.18E-14	1.78E-10
Fibronectin leucine rich transmembrane protein 2	Flrt2	-1.1175	1.18E-05	0.0113
BCL2/adenovirus E1B interacting protein 3	Bnip3	-1.5543	3.02E-06	0.0045
Hyaluronoglucosaminidase 1	Hyal1	-2.0573	4.86E-08	1.02E-04
Tropomodulin 1	Tmod1	-3.4012	4.96E-05	0.0311

List of the DEGs in macrophage cells after exposure to *C. neoformans*. m.value: numeric vector of fold-change on $\log_2$ scale for each gene between the two groups compared (*C. neoformans* x control). p.value: numeric vector of p-value. q.value: numeric vector of q-value calculated based on the p-value using FDR of 0.1.
