# Supplementary material for: Transcriptomic analysis reveals that mTOR pathway can be modulated in macrophage cells by the presence of cryptococcal cells
Source: Genet Mol Biol. 2021 Aug 2;44(3):e20200390. doi: 10.1590/1678-4685-GMB-2020-0390 (PMC8341293; doi:10.1590/1678-4685-GMB-2020-0390)
Supplement: Table S4 - [file 1415-4757-GMB-44-3-e20200390-s6.pdf]

Genetics and Molecular Biology
https://doi.org/10.1590/1678-4685-GMB-2020-0390

Supplementary Material to "Transcriptomic analysis reveals that mTOR pathway can be modulated in macrophage cells by the presence of cryptococcal cells"

Table S4 - Score and number of nodes for the two main networks.

***C. gattii* network**

	Score	Nodes
Cluster 1	35.008	378
Cluster 2	21.41	371
Cluster 3	19.523	283
Cluster 4	19.353	215
Cluster 5	15.495	186
Cluster 6	8.777	94
Cluster 7	6.893	28
Cluster 8	3.59	78

***C. neoformans* network**

	Score	Nodes
Cluster 1	41.459	508
Cluster 2	21.804	439
Cluster 3	16.083	156
Cluster 4	14.692	156
Cluster 5	12.855	110
Cluster 6	9.766	47
Cluster 7	5.639	108
Cluster 8	4.286	63
Cluster 9	3.862	29
Cluster 10	3.375	8
Cluster 11	2.75	24
Cluster 12	2.071	14

The analysis of the networks in terms of the major clusters or module composition was performed using the program Molecular Complex Detection (MCODE). The score cutoff was 2. *C. gatti* network present 8 clusters and *C. neoformans* network present 12 cluster.
