# Supplementary material for: Transcriptomic analysis reveals that mTOR pathway can be modulated in macrophage cells by the presence of cryptococcal cells
Source: Genet Mol Biol. 2021 Aug 2;44(3):e20200390. doi: 10.1590/1678-4685-GMB-2020-0390 (PMC8341293; doi:10.1590/1678-4685-GMB-2020-0390)
Supplement: Table S5 - [file 1415-4757-GMB-44-3-e20200390-s7.pdf]

Genetics and Molecular Biology
https://doi.org/10.1590/1678-4685-GMB-2020-0390

Supplementary Material to "Transcriptomic analysis reveals that mTOR pathway can be modulated in macrophage cells by the presence of cryptococcal cells"

Table S5 - Bioprocess *C. gattii* network.

GO-ID	Description	Total Nodes	Up-regulated nodes	Down-regulated nodes	FDR-corrected P value
51301	Cell division	105	Bub1b<br>Ube2c<br>Cdca2	-	3.96E-87
16310	Phosphorylation	93	Bub1b	Pfkl<br>Pdk1<br>Plk2	3.08E-32
23052	Signaling	217	Gucala<br>Fcgr1	Pten<br>Vav3<br>Pdk1	3.78E-30
50896	Response to stimulus	163	Gucala<br>Foxm1<br>Fcgr1	Pfkl<br>Pten<br>Pdk1<br>Egln1<br>Ddit4	1.88E-26
30154	Cell differentiation	120	Foxm1	Pten<br>Bnip3	2.67E-21
42127	Regulation of cell proliferation	70	Foxm1	Pten<br>Cd274	7.82E-20
2376	Immune system process	69	Fcgr1	Ndrg1	8.55E-18
22607	Cellular component assembly	61	Tuba4a	Pfkl<br>Pdk1<br>Vav3	1.07E-17
42325	Regulation of phosphorylation	47	-	Vav3	5.36E-15

GO-ID	Description	Total Nodes	Up-regulated nodes	Down-regulated nodes	FDR-corrected P value
2682	Regulation of immune system process	46	Fcgr1	Cd274	2.46E-14
10468	Regulation of gene expression	126	Foxm1	Pten<br>Nr1d1	2.67E-14
10941	Regulation of cell death	59	Fcgr1	Pten<br>Bnip3	1.26E-12
6464	Protein modification process	81	Bub1b<br>Ube2c	Pten<br>Pdk1<br>Plk2	6.99E-12
10646	Regulation of cell communication	66	Foxm1	Pfkl<br>Pten<br>Vav3<br>Ddit4	6.42E-11
30030	Cell projection organization	39	-	Pten<br>Vav3	5.24E-09
45595	Regulation of cell differentiation	47	-	Pten	9.10E-09
48583	Regulation of response to stimulus	41	Foxm1<br>Fcgr1	-	1.69E-08
16477	Cell migration	31	-	Pten<br>Vav3	5.53E-08
51049	Regulation of transport	43	Fcgr1	Pfkl<br>Pten	2.72E-07
1775	Cell activation	27	-	Ndrg1	2.55E-05
50865	Regulation of cell activation	22	-	Cd274	4.66E-05
16265	Death	39	Bub1b	Pten<br>Ddit4<br>Bnip3	1.27E-04
16192	Vesicule mediated transport	32	Fcgr1	Vav3	2.74E-02
6952	Defense response	30	Fcgr1	-	3.03E-02

GO-ID	Description	Total Nodes	Up-regulated nodes	Down-regulated nodes	FDR-corrected P value
16043	Cellular component organization	156	Tuba4a<br>Bub1b<br>Espl1<br>Ube2c<br>Cdca2	Pten<br>Vav3	1.40E-80
23034	Intracellular signaling pathway	46	-	Vav3	1.00E-10
51128	Regulation of cellular component organization	31	-	Pten	2.00E-09
43412	Macromolecule modification	48	Bub1b<br>Ube2c	Pten<br>Plk2	6.58E-07
45941	Positive regulation of transcription	30	Foxm1	-	1.33E-05
30031	Cell projection assembly	11	-	Vav3	2.20E-04
35466	Regulation of signaling pathway	27	Foxm1	Pten<br>Vav3	1.09E-02
10628	Positive regulation of gene expression	52	Foxm1	-	2.54E-22
42981	Regulation of apoptosis	45	Fcgr1	Pten	6.01E-16
6915	Apoptosis	22	-	Pten<br>Ddit4	7.17E-03
31344	Regulation of cell projection organization	12	-	Pten	1.92E-02
6096	Glycolysis	14	-	Pfkl	1.53E-13
19882	Antigen processing and presentation	11	Fcgr1	-	7.71E-08
2694	Regulation of leukocyte activation	16	-	Cd274	8.48E-07
6954	Inflammatory response	17	Fcgr1	-	4.70E-06
2443	Leukocyte mediated immunity	12	Fcgr1	-	5.50E-06
43687	Post-translation protein	35	-	Pten	1.94E-09

GO-ID	Description	Total Nodes	Up-regulated nodes	Down-regulated nodes	FDR-corrected P value
	modification				
48870	Cell motility	15	-	Pten<br>Vav3	6.24E-04
30258	Lipid modification	22	-	Pten	3.28E-26
43067	Regulation of programmed cell death	28	-	Pten	2.04E-10
6006	Glucose metabolic process	13	-	Pfkl<br>Pdk1	7.54E-07
30334	Regulation of cell migration	13	-	Pten	2.14E-06
16311	Dephosphorylation	13	-	Pten	1.80E-05
7166	Cell surface receptor linked signaling pathway	28	-	Vav3	3.03E-04
19318	Hexose/carbohydrate metabolic process	18	-	Pfkl	6.47E-28
46835	Carbohydrate phosphorylation	8	-	Pfkl	8.63E-16
16043	Cellular component organization	29	-	Vav3	7.73E-09
51171	Regulation of nitrogen compound metabolic process	28	-	Vav3	7.22E-07
6796	Phosphate metabolic process	33	Bub1b	Pten	3.88E-15
51270	Regulation of cellular component movement	11	-	Pten	3.90E-05
6996	Organelle organization	36	Bub1b<br>Espl1<br>Ube2c	Vav3	4.54E-17

List of the selected bioprocesses from the *C. gattii* network and clusters. The analysis was performed using the BINGO 2.44 plugin, in the Cytoscape 2.8.3 software.
