# Supplementary material for: Transcriptomic analysis reveals that mTOR pathway can be modulated in macrophage cells by the presence of cryptococcal cells
Source: Genet Mol Biol. 2021 Aug 2;44(3):e20200390. doi: 10.1590/1678-4685-GMB-2020-0390 (PMC8341293; doi:10.1590/1678-4685-GMB-2020-0390)
Supplement: Table S6 - [file 1415-4757-GMB-44-3-e20200390-s8.pdf]

Genetics and Molecular Biology
https://doi.org/10.1590/1678-4685-GMB-2020-0390

Supplementary Material to "Transcriptomic analysis reveals that mTOR pathway can be modulated in macrophage cells by the presence of cryptococcal cells"

Table S6 - Bioprocess *C. neoformans* network.

GO-ID	Description	Total Nodes	Up-regulated nodes	Down-regulated nodes	FDR-corrected P value
6974	Response to DNA damage stimulus	59	H2afx	-	8.77E-32
6950	Response to stress	115	H2afx<br>Txnip<br>Myo1f	Ddit4<br>Pdk1	3.19E-31
50896	Response to stimulus	163	H2afx<br>Txnip<br>Myo1f	Ddit4<br>Pdk1<br>Pfkl	8.71E-28
10468	Regulation of gene expression	144	Txnip	Kdm3a<br>Jmjd1c<br>Ell	8.10E-24
7010	Cytoskeleton organization	50	Kif11	Tmod1	1.40E-22
30154	Cell differentiation	118	Cenpf	Bnip3<br>Kdm3a<br>Tmod1	4.12E-21
43412	Macromolecule modification	94	Aurka<br>Ube2c	P4ha1<br>Pdk1	4.30E-18
16310	Phosphorylation	70	Aurka	Pdk1<br>Pfkl	1.31E-16
10941	Regulation of cell death	63	Cenpf<br>Txnip	Bnip3	1.33E-15
8219	Cell death	55	-	Bnip3<br>Ddit4	5.58E-15

GO-ID	Description	Total Nodes	Up-regulated nodes	Down-regulated nodes	FDR-corrected P value
				Hyal1	
71103	DNA conformation change	24	H2afx<br>Ncapd2	-	7.93E-15
6096	Glycolysis	17	-	Pfkl	3.91E-12
2376	Immune system process	59	Myo1f	Ndrg1	6.18E-12
10467	Gene expression	117	Txnip	Jmjd1c<br>Kdm3a<br>Ell	1.28E-10
32386	Regulation of intracellular transport	13	Cenpf	-	6.26E-06
46835	Carbohydrate phosphorylation	8	-	Pfkl	1.23E-04
6310	DNA recombination	12	H2afx	-	6.29E-03
32880	Regulation of protein localization	14	Cenpf	-	2.16E-02
16567	Protein ubiquitination	7	Ube2c	-	4.65E-03
6468	Protein aminoacid phosphorylation	13	Aurka	-	1.75E-02
7049	Cell cycle	182	Cdkn3<br>Fam83d<br>Aurka<br>Ube2c<br>Kif11<br>H2afx<br>Ncapd2	-	0.0000E-100
48285	Organelle fission	109	Fam83d<br>Aurka<br>Ube2c<br>Kif11<br>Ncapd2	-	0.0000E-100
51726	Regulation of cell cycle	44	Cdkn3	-	1.37E-27

GO-ID	Description	Total Nodes	Up-regulated nodes	Down-regulated nodes	FDR-corrected P value
			Cenpf		
43067	Regulation of programmed cell death	37	Cenpf	-	6.26E-06
16043	Cellular component organization	131	Fam83d H2afx	Pfkl	5.65E-47
6007	Glucose catabolic process	15	-	Pfkl	1.11E-10
10646	Regulation of cell communication	37	-	Ddit4 Pfkl	3.88E-04
6091	Generation of precursor metabolites and energy	17	-	Pfkl	1.19E-02
9617	Response to bacterium	11	Myo1f	-	4.42E-04
6955	Immune response	13	Myo1f	-	4.29E-03
6952	Defense response	14	Myo1f	-	7.92E-03
22607	Cellular component assembly	23	H2afx	-	7.10E-10
51707	Response to other organism	14	Myo1f	-	1.35E-07
2682	Regulation of immune system process	13	Myo1f	-	1.47E-04
6996	Organelle organization	16	-	Tmod1	3.08E-07
19318	Hexose metabolic process	25	-	Pfkl	1.35E-27
51049	Regulation of transport	16	-	Pfkl	4.79E-06
1775	Cell activation	9	-	Ndrg1	2.36E-02
43687	Pos-translation protein modification	20	-	Pdk1	1.29E-16
23052	Signaling	21	-	Pdk1	1.38E-07
5996	Monosaccharide metabolic process	6	-	Pdk1	4.83E-07
6464	Protein modification process	14	-	Pdk1	5.53E-08

List of the selected bioprocesses from the *C. neoformans* network and clusters. The analysis was performed using the BINGO 2.44 plugin, in the Cytoscape 2.8.3 software.
